# Tissue factor regulates autophagy in pulmonary artery endothelial cells from chronic thromboembolic pulmonary hypertension rats via the p38 MAPK-FoxO1 pathway

**DOI:** 10.1186/s12931-024-02886-z

**Published:** 2024-06-28

**Authors:** Dawen Wu, Yi Lin, Minxia Yang, Hongli Li, Wenfeng Wang, Qiuxia Wu, Maohe Chen, Nan Shao, Chaosheng Deng

**Affiliations:** 1 Department of Respiratory and Critical Care Medicine, First Affiliated Hospital of Fujian Medical University, Fuzhou, 350005 China; 2 Department of Respiratory and Critical Care Medicine, National Regional Medical Center, Binhai Campus of the First Affiliated Hospital, Fujian Medical University, Fuzhou, 350212 China; 3 Institute of Respiratory Disease, Fujian Medical University, Fuzhou, 350005 China; 4 School of Basic Medical Sciences, Fujian Medical University, Fuzhou, 350122 China; 5 Division of Critical Care Medicine, First Affiliated Hospital of Fujian Medical University, Fuzhou, 350005 China

**Keywords:** Tissue factor, Forkhead box transcription factor O-1, Autophagy, Mitogen-activated protein kinase, Pulmonary artery endothelial cells, Chronic thromboembolic pulmonary hypertension, Coimmunoprecipitation

## Abstract

**Aims:**

To detect the expression of autophagy components, p38 MAPK (p38) and phosphorylated forkhead box transcription factor O-1 (pFoxO1) in pulmonary vascular endothelial cells of chronic thromboembolic pulmonary hypertension (CTEPH) rats and to investigate the possible mechanism through which tissue factor (TF) regulates autophagy.

**Methods:**

Pulmonary artery endothelial cells (PAECs) were isolated from CTEPH (CTEPH group) and healthy rats (control group (ctrl group)) which were cocultured with TF at different time points including 12 h, 24 h, 48 h and doses including 0 nM,10 nM, 100 nM, 1µM, 10µM, 100µM and cocultured with TFPI at 48 h including 0 nM, 2.5 nM, 5 nM. The expression of forkhead box transcription factor O-1 (FoxO1), pFoxO1, p38, Beclin-1 and LC3B in PAECs was measured. Coimmunoprecipitation (co-IP) assays were used to detect the interaction between FoxO1 and LC3.

**Results:**

The protein expression of p-FoxO1/FoxO1 was significantly lower in the CTEPH groups (cocultured with TF from 0 nM to 100 µM) than in the ctrl group at 12 h, 24 h, and 48 h (*P* < *0.05*) and was significantly lower in the CTEPH groups (cocultured with TFPI from 0 nM to 5 nM) than in the ctrl group at 48 h (*P* < *0.05*). The protein expression of p38 in the CTEPH groups treated with 0 nM, 10 nM, 100 nM or 1 µM TF for 48 h significantly increased than ctrl groups (*P* < *0.05*) and was significantly increased in the CTEPH groups (cocultured with TFPI concentration from 0 nM to 5 nM) than in the ctrl group at 48 h (*P* < *0.05*). The protein expression of Beclin1 at the same concentration (cocultured with TF from 0 nM to 100 µM) was significantly lower in the CTEPH groups than ctrl groups after 24 h and 48 h (*P* < *0.05*) and was significantly decreased in the CTEPH groups (cocultured with TFPI concentration from 2.5 nM to 5 nM) than in the ctrl group at 48 h (*P* < *0.05*). The protein expression of LC3-II/LC3-I at the same concentration (cocultured with TF 0 nM, 1 µM, 10 µM, and 100 µM) was significantly lower in the CTEPH than in the ctrl groups after 12 h (*P* < *0.05*) and was significantly lower in the CTEPH groups (cocultured with TFPI concentration from 0 nM to 5 nM) than in the ctrl group at 48 h (*P* < *0.05*). There were close interactions between FoxO1 and LC3 in the control and CTEPH groups at different doses and time points.

**Conclusion:**

The autophagic activity of PAECs from CTEPH rats was disrupted. TF, FoxO1 and p38 MAPK play key roles in the autophagic activity of PAECs. TF may regulate autophagic activity through the p38 MAPK-FoxO1 pathway.

## Background

Chronic thromboembolic pulmonary hypertension (CTEPH) is a rare pulmonary vascular disease secondary to pulmonary thromboembolism resulting in pulmonary hypertension [1]. The pathophysiology of this disease is complex and includes thrombosis, unresolved clots and vascular remodeling. Pulmonary artery endothelial cells (PAECs) dysfunction is a characteristic of vascular remodeling that mainly manifests as endothelial mesenchymal transition (EndMT) accompanied by hyperproliferation and impaired autophagic function [2].

Thrombotic factor tissue factor (TF) is a membrane-bound protein that contributes to the initiation of the extrinsic coagulation pathway and plays a key role in the development to pulmonary embolism. Our previous study revealed that the expression level of the thrombotic factor TF in the blood was increased in patients with CTEPH [3]. In addition, TF was shown to increase in the pulmonary artery of a CTEPH rat model [4]. Autophagy is dysregulated in a rat model of CTEPH, and indicators of autophagy, including Beclin-1 and microtubule-associated protein 1 light chain (LC3), are decreased in the pulmonary artery [4]. Hu et al. found that reduced autophagy was involved in CTEPH-induced HPAEC dysfunction [5]. Forkhead box transcription factor O-1 (FoxO1), which is a member of the Foxo family [6] and a downstream signal of mitogen-activated protein kinase (MAPK) [7], plays critical roles in the cell cycle, proliferation, apoptosis, and tumorigenesis and regulates endothelial cell autophagy. Our previous study revealed that FoxO1 expression was reduced in the pulmonary artery of a CTEPH rat model [8]. Autophagy was activated in monocrotaline-pulmonary arterial hypertension (PAH) rats and elevation of cytosolic FoxO1 could stimulate autophagy activation of pulmonary arterial smooth muscle cells from PAH ratsl [9]. TF can promote vascular remodeling and upregulate the expression of vascular endothelial growth factor through MAPK [10]. Therefore, we speculated that TF may regulate autophagy capacity via the p38 MAPK-FoxO1 signaling pathway in CTEPH rats. However, the underlying mechanism was not clear.

Therefore, we aimed to detect the expression of autophagy, p38 MAPK (p38) and pFoxO1 in PAECs from CTEPH rats and then investigate the possible mechanism which TF regulates autophagy.

## Methods

### Cell culture and groups

**Fig. 1:**
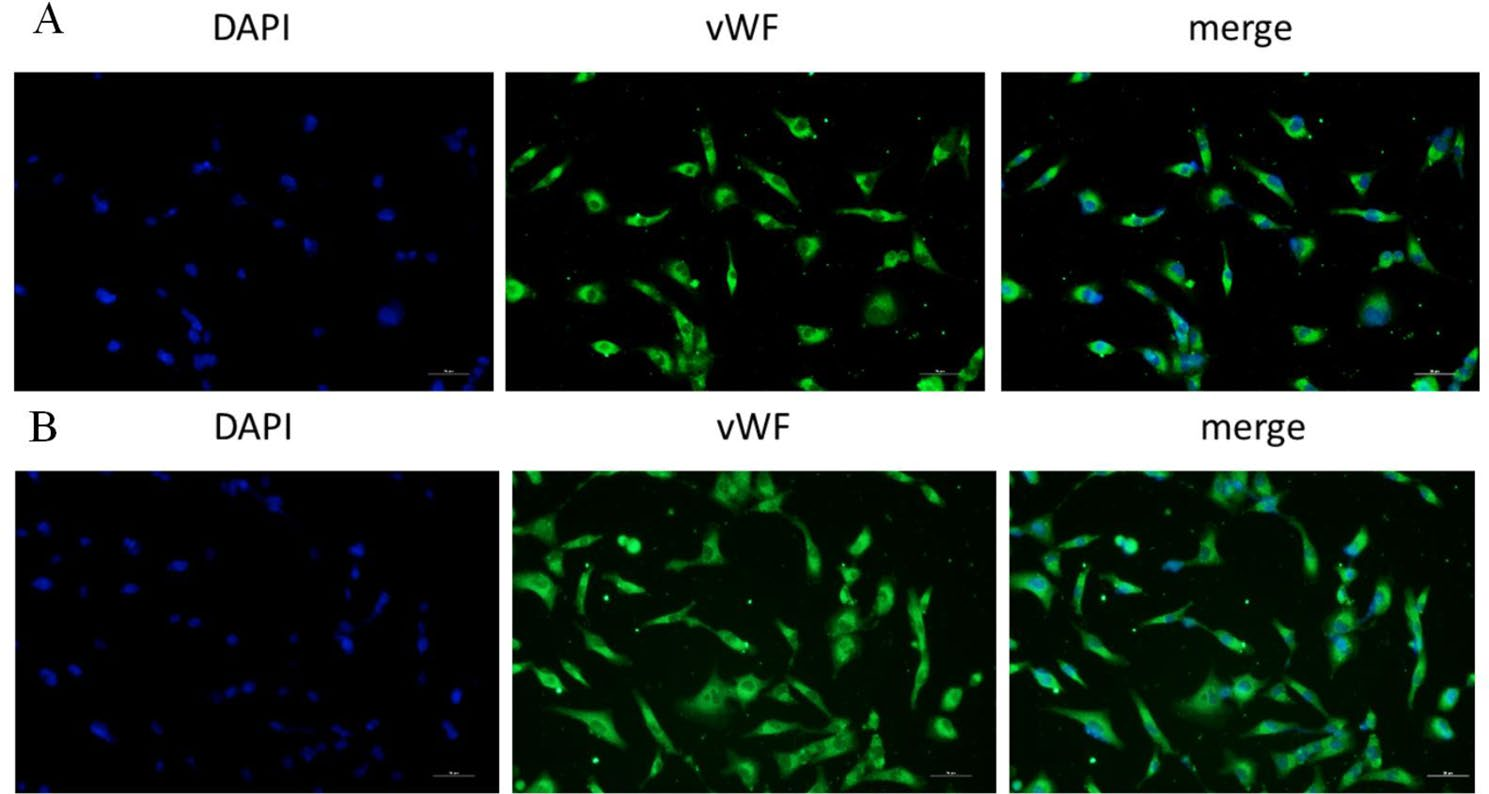
Pulmonary artery endothelial cells (PAECs). Notes: (**A**) PAECs from healthy SD rats, the positive cell rate for vWF was 53.8%; (**B**) PAECs from CTEPH rats, the positive cell rate for vWF was 57.4%

All animal procedures were performed in accordance with the Animal Ethics Committee of Fujian Medical University Institutional Animal Care (SYXK 2012-0001) and Care and Use of Laboratory Animals (NIH, Bethesda, MD, USA) guidelines. The CTEPH rat model was established according to previous methods [4]. PAECs were isolated from CTEPH or healthy male Sprague Dawley (SD) rats that were cultured in culture medium supplemented with 10% fetal bovine serum in a cell culture box containing 5% CO_2_ at 37 °C. The cells were removed from the culture box when they had proliferated to 80-90%, and the cells were subsequently washed twice gently with precooled PBS (Nan Jing Sun Shine Biotechnology). After fixation at room temperature for 10 min with 4% paraformaldehyde, the cells were incubated with an anti-vWF antibody (1:2000; ab6994; Abcam) at room temperature for 1 h and 100 ng/mL DAPI (Thermo Fisher) at room temperature for 10 min. Then, anti-fluorescence quenching solution was added to these cells, and images were taken directly under a fluorescence microscope to identify PAECs (Fig. 1). PAECs were isolated from CTEPH and healthy rats (control group (ctrl group)) which were cocultured with TF in different time points including 12 h (h), 24 h, 48 h and doses including 0 nm (nM),10 nM,100 nM,1micrometre (µM), 10µM,100µM. At the same time, PAECs were isolated from CTEPH and ctrl group which were cocultured with tissue factor pathway inhibitor (TFPI) at 48 h and different doses including 0 nM, 2.5nM and 5nM.

### Western blot

Western blotting was used to analyze the expression of pFoxO1, FoxO1, p38, Beclin-1 and LC3B. Cells were lysed with RIPA buffer (P0013B; Beyotime Biotechnology, Shanghai, China). After the cells were lysed, the protein concentration was measured with a BCA assay (P0011; Beyotime Biotechnology, Shanghai, China), and 30 µg of protein was subsequently loaded onto a sodium dodecyl sulfate‒polyacrylamide gel electrophoresis (SDS‒PAGE) gel. After electrophoresis, blocking in 5% skim milk in TBST solution, the blots were probed with the following primary antibodies: pFoxO1 (1:1000, ab283583, Abcam), FoxO1 (1:1000, ab179450, Abcam), p38 (1:1000, ab170099, Abcam), Beclin-1 (1:1000, ab302669, Abcam), and LC3B (1:1000, ab192890, Abcam). Anti-β-actin (1:1000, ab8226, Abcam) was used as a loading control.

### Coimmunoprecipitation

Aimed to detect the interaction between FoxO1 and LC3, we used the Co-Immunoprecipitation (Co-IP) assays. Cells were lysed with RIPA buffer and protein concentration was measured with the BCA assay. The volume of each IP experimental sample was 500ul, and 30ul of each sample was reserved as experimental input. Protein A/G bead (50 ul 50% bead concentration required per IP sample) was prepared. The supernatant was transferred to a new centrifuge tube and protein A/G bead was removed. Each well was incubated with 5 ug FoxO1 antibody, mixed with vertical rotation and slowly rotating overnight at 4 °C. The next morning, add 50 ul of protein A/G bead (containing 50% concentration) to each protein sample. The IP and input samples were subjected to SDS-PAGE electrophoresis. About 10 µl sample was added to PAGE gel for electrophoresis.1 ml of the luminescent liquid was added to the film, and exposed on an exposure apparatus.

### Statistical analysis

SPSS 27.0 (IBM, Armonk, NY, USA) software was used for the statistical analysis. Numerical parameters with a normal Gaussian distribution are expressed as the mean ± standard deviation (SD). Parameters among time points within each group or subgroup were compared by analysis of variance. *P* values *< 0.05* were considered to indicate significant differences.

## Results

### The protein expression of p-FoxO1/FoxO1 in PAECs decreased after stimulation of TF

**Fig. 2:**
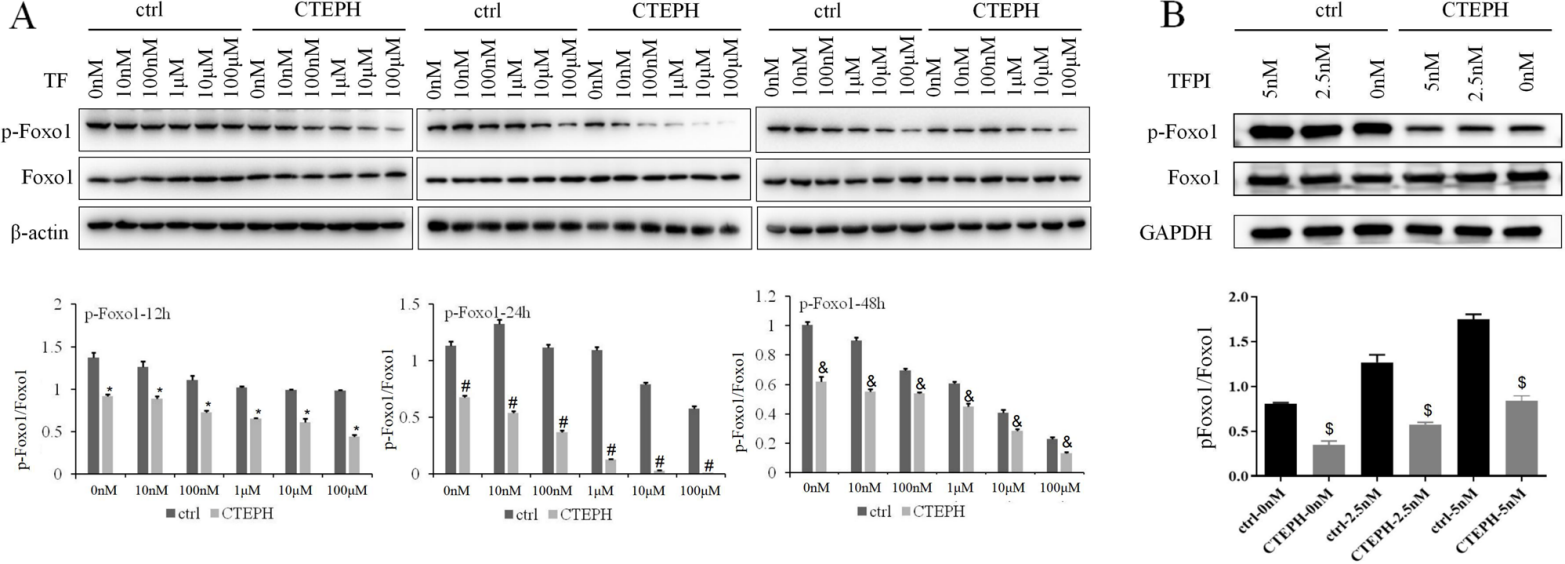
The protein expression of p-FoxO1/FoxO1 in PAECs decreased after stimulation of TF. Notes: (**A**): In the ctrl group, PAECs from healthy SD rats were cocultured with TF; in the CTEPH group, PAECs from CTEPH rats were cocultured with TF. ^*^P < *0.05*, the protein expression of p-FoxO1/FoxO1 was significantly lower at the same concentration of TF in the CTEPH groups than the ctrl groups at 12 h. ^#^*P* < *0.05*, the protein expression of p-FoxO1/FoxO1 was significantly lower after treatment with the same concentration of TF between in the CTEPH groups than the ctrl groups at 24 h. ^&^*P* < *0.05*, the protein expression of p-FoxO1/FoxO1 was significantly lower after treatment with the same concentration of TF in the CTEPH groups than the ctrl groups at 48 h. (**B**): In the ctrl group, PAECs from healthy SD rats were cocultured with TFPI; in the CTEPH group, PAECs from CTEPH rats were cocultured with TFPI. ^$^*P* < *0.05*, the protein expression of p-FoxO1/FoxO1 was significantly lower after treatment with the same concentrations of TFPI in the CTEPH groups than the ctrl groups at 48 h

The protein expression of p-FoxO1/FoxO1 in the ctrl group gradually decreased with increasing TF concentration from 0 nM to 100 µM (*P* < *0.05*) after 48 h. There was a decreasing trend in the protein expression of p-FoxO1/FoxO1 in the ctrl group depending on the TF concentration (0 nM, 10 nM, 100 nM or 1 µM) after 12 h. There was a decreasing trend in the protein expression of p-FoxO1/FoxO1 in the ctrl group depending on the TF concentration (10 nM, 1 µM, 10 µM or 100 µM) after 24 h. The protein expression of p-FoxO1/FoxO1 in CTEPH rats also gradually decreased in response to TF concentrations ranging from 0 to 10 nm, 100 nm and 1 μm after 24 h (*P* < 0.05). The trend toward a decrease in the protein expression of p-FoxO1/FoxO1 in CTEPH rats was dependent on the TF concentration (10 nM, 100 nM, 10 µM or 100 µM) after 12 h. The trend toward a decrease in the protein expression of p-FoxO1/FoxO1 in CTEPH rats was dependent on the TF concentration (0 nM, 100 nM, 10 µM, 10 µM or 100 µM) after 48 h. Similarly, the protein expression of p-FoxO1/FoxO1 was significantly lower in the CTEPH groups (from 0 nM to 100 µM) than in the ctrl group at 12 h, 24 h, and 48 h (*P* < *0.05*) (Fig. 2A). At the same time, we could see that there was a increasing trend in the protein expression of p-FoxO1/FoxO1 in the ctrl group/CTEPH group depending on the TFPI concentration from 0 nM to 5 nM after 48 h. However, the protein expression of p-FoxO1/FoxO1 was significantly lower in the CTEPH groups (TFPI concentration from 0 nM to 5 nM) than in the ctrl group at 48 h (*P* < *0.05*) (Fig. 2B).The expression of p-FoxO1/FoxO1 may be negatively regulated by TF and embolism.

### The protein expression of p38 in PAECs increased after stimulation of TF

**Fig. 3:**
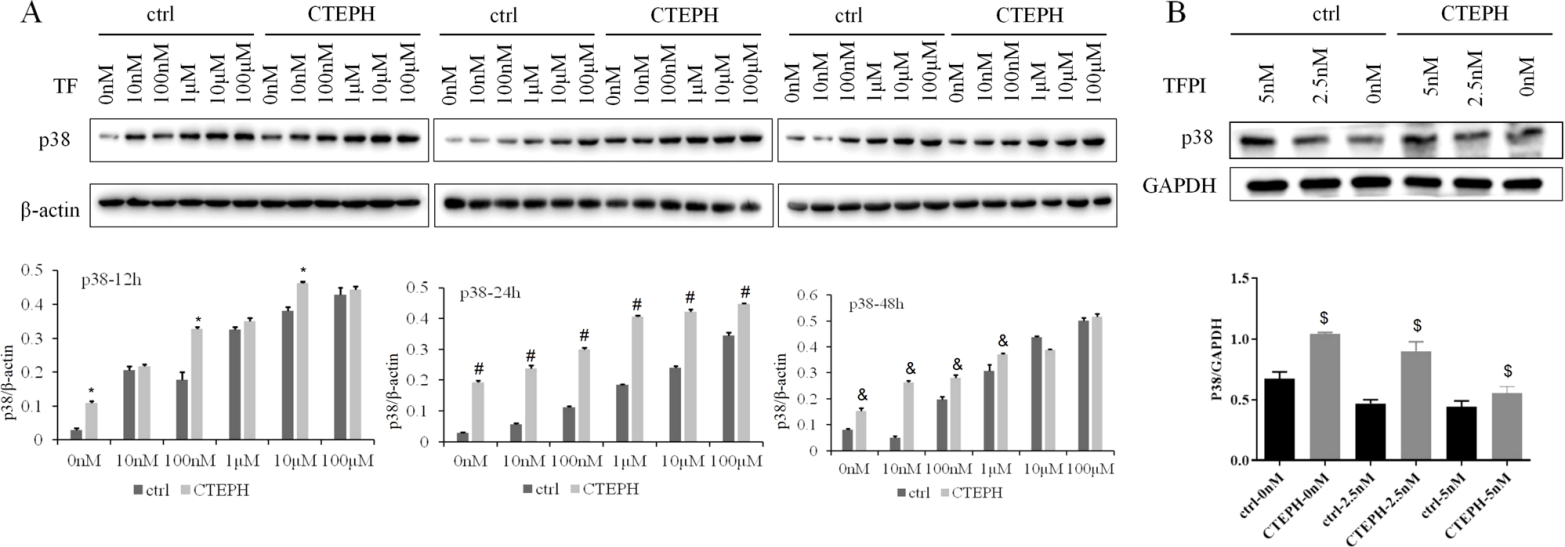
The protein expression of p38 in PAECs increased after stimulation of TF. Notes: (**A**): In the ctrl group, PAECs from healthy SD rats were cocultured with TF; in the CTEPH group, PAECs from CTEPH rats were cocultured with TF. ^*^P < *0.05*, the protein expression of p38 was significantly increased at the same concentration of TF in the CTEPH groups than the ctrl groups at 12 h. ^#^*P* < *0.05*, the protein expression of p38 was significantly increased after treatment with the same concentration of TF in the CTEPH groups than the ctrl groups at 24 h. ^&^*P* < *0.05*, the protein expression of p38 was significantly increased after treatment with the same concentration of TF in the CTEPH groups than the ctrl groups at 48 h. (**B**): In the ctrl group, PAECs from healthy SD rats were cocultured with TFPI; in the CTEPH group, PAECs from CTEPH rats were cocultured with TFPI. ^$^*P* < *0.05*, the protein expression of p38 was significantly increased after treatment with the same concentrations of TFPI in the CTEPH groups than the ctrl groups at 48 h

The protein expression of p38 in the ctrl group gradually increased with increasing TF concentration from 0 nM to 100 µM (*P* < *0.05*) after 24 h. The increase in the protein expression of p38 in the ctrl group was dependent on the TF concentration (from 0 nM to 100 µM) at 12 h and 48 h. The protein expression of p38 in CTEPH rats also gradually increased with increasing TF concentration from 0 nM to 100 µM (*P* < *0.05*) after 24 h. The increase in the protein expression of p38 in CTEPH rats was dependent on the concentration of TF, which ranged from 0 nM to 100 µM after 12 h and 48 h. At the same concentration (from 0 nM to 100 µM), the protein expression of p38 was significantly greater in the CTEPH groups than ctrl groups after 24 h (*P* < *0.05*). The protein expression of p38 in the CTEPH groups treated with 0 nM, 100 nM, 1 µM or 10 µM TF for 12 h significantly increased than ctrl groups (*P* < *0.05*). The protein expression of p38 in the CTEPH groups treated with 0 nM, 10 nM, 100 nM or 1 µM TF for 48 h significantly increased than ctrl groups (*P* < *0.05*) (Fig. 3A). At the same time, we could see that there was a decreasing trend in the protein expression of p38 in the ctrl group/CTEPH group depending on the TFPI concentration from 0 nM to 5 nM after 48 h. However, the protein expression of p38 was significantly increased in the CTEPH groups (TFPI concentration from 0 nM to 5 nM) than in the ctrl group at 48 h (*P* < *0.05*) (Fig. 3B). The expression of p38 may be positively regulated by TF and embolism.

### The protein expression of Beclin1 in PAECs decreased after stimulation of TF

**Fig. 4:**
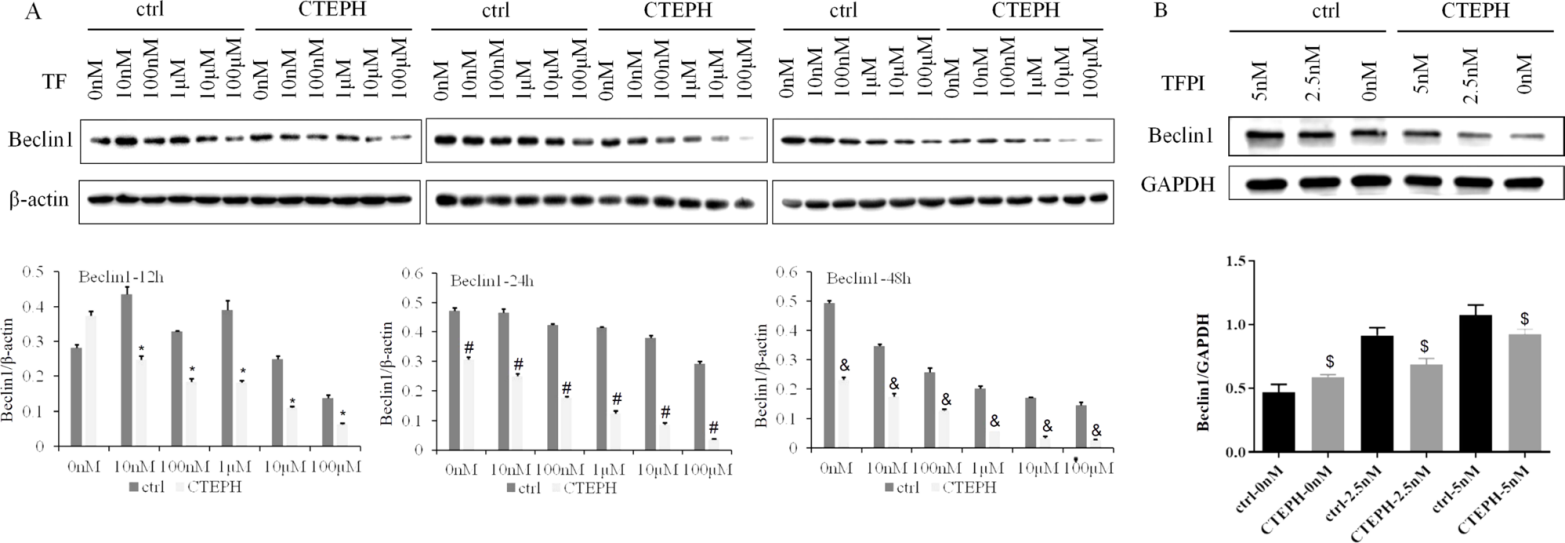
The protein expression of Beclin1 in PAECs decreased after stimulation of TF. Notes: (**A**): In the ctrl group, PAECs from healthy SD rats were cocultured with TF; in the CTEPH group, PAECs from CTEPH rats were cocultured with TF. ^*^P < *0.05*, the protein expression of Beclin1 was significantly decreased at the same concentration of TF in the CTEPH groups than the ctrl groups at 12 h. ^#^*P* < *0.05*, the protein expression of Beclin1 was significantly decreased at the same concentration of TF in the CTEPH groups than the ctrl groups at 24 h. ^&^*P* < *0.05*, the protein expression of Beclin1 was significantly decreased at the same concentration of TF in the CTEPH groups than the ctrl groups at 48 h. (**B**): In the ctrl group, PAECs from healthy SD rats were cocultured with TFPI; in the CTEPH group, PAECs from CTEPH rats were cocultured with TFPI. ^$^*P* < *0.05*, the protein expression of Beclin1 was significantly lower after treatment with the same concentrations of TFPI in the CTEPH groups than the ctrl groups at 48 h

The protein expression of Beclin1 in the ctrl group gradually decreased with increasing TF concentration from 0 nM to 100 µM after 48 h (*P* < *0.05*). The change in the protein expression of Beclin1 in the ctrl group decreased with increasing TF concentration from 10 nM to 100 µM after 12 h and 24 h. The protein expression of Beclin1 in CTEPH rats also gradually decreased with increasing TF concentration from 0 nM to 100 µM (*P* < *0.05*) at 24 h and 48 h. The decrease in the protein expression of Beclin1 in CTEPH rats was dependent on the concentration of TF, which ranged from 0 nM to 100 µM after 24 h and 48 h. The protein expression of Beclin1 at the same concentration (from 0 nM to 100 µM) was significantly lower in the CTEPH groups than ctrl groups after 24 h and 48 h (*P* < *0.05*). The protein expression of Beclin1 from CTEPH groups in the TF concentration of 10 nM, 100 nM, 1µM, 10µM and 100µM in 12 h were also significantly decreased than ctrl groups (*P* < *0.05*) (Fig. 4A). At the same time, we could see that there was a increasing trend in the protein expression of Beclin1 in the ctrl group/CTEPH group depending on the TFPI concentration from 0 nM to 5 nM after 48 h. However, the protein expression of Beclin1 was significantly decreased in the CTEPH groups (TFPI concentration from 2.5 nM to 5 nM) than in the ctrl group at 48 h (*P* < *0.05*) (Fig. 4B). The expression of Beclin1 may be negatively regulated by TF and embolism.

### The expression of LC3-II/LC3-I in PAECs decreased after stimulation of TF

**Fig. 5:**
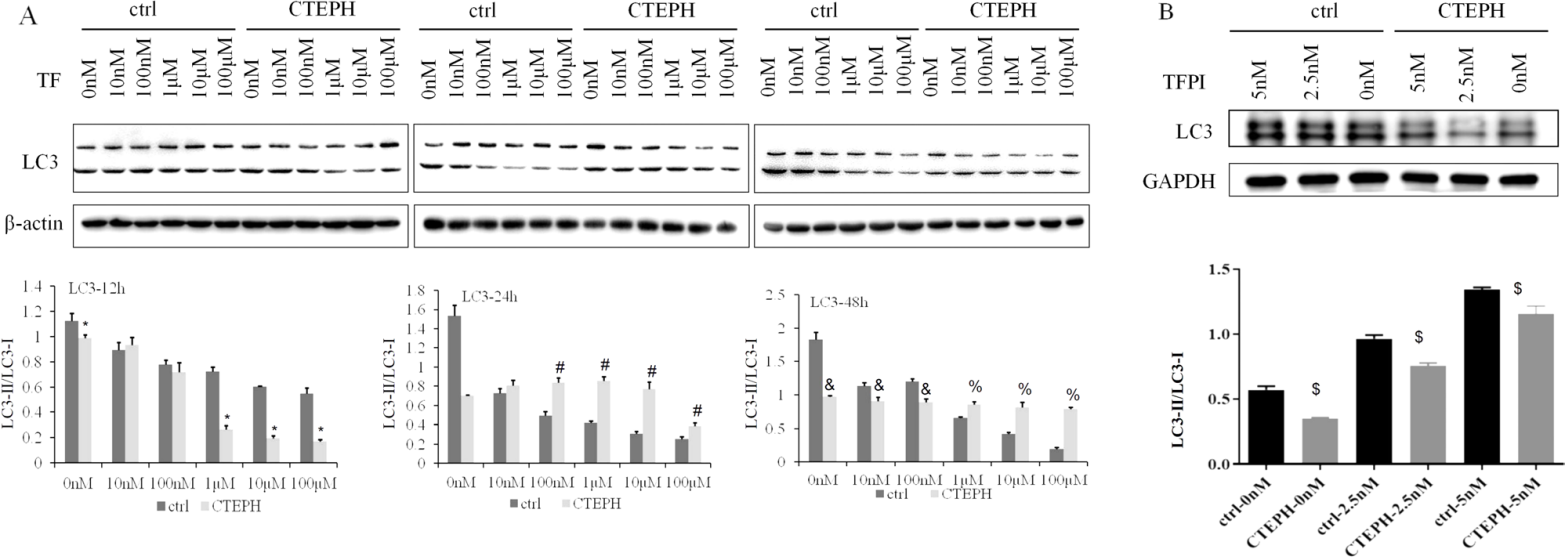
The expression of LC3-II/LC3-I in PAECs decreased after stimulation of TF. Notes: (**A**): In the ctrl group, PAECs from healthy SD rats were cocultured with TF; in the CTEPH group, PAECs from CTEPH rats were cocultured with TF. ^*^P < *0.05*, the expression of LC3-II/LC3-I significantly decreased at the same concentration of TF in the CTEPH groups than the ctrl groups at 12 h. ^#^*P* < *0.05*, the expression of LC3-II/LC3-I significantly increased at the same concentration of TF in the CTEPH groups than the ctrl groups at 24 h. ^&^*P* < *0.05*, the expression of LC3-II/LC3-I significantly decreased at the same concentration of TF in the CTEPH groups than the ctrl groups at 48 h. ^%^*P* < *0.05*, the expression of LC3-II/LC3-I was significantly increased at the same concentration of TF in the CTEPH groups than the ctrl groups at 48 h. (**B**): In the ctrl group, PAECs from healthy SD rats were cocultured with TFPI; in the CTEPH group, PAECs from CTEPH rats were cocultured with TFPI. ^$^*P* < *0.05*, the protein expression of Beclin1 was significantly lower after treatment with the same concentrations of TFPI in the CTEPH groups than the ctrl groups at 48 h

There was a decreasing trend in the expression of LC3-II/LC3-I in the ctrl group depending on the TF concentration ranging from 0 nM to 100 µM at 12 h, 24 h and 48 h. The trend toward a decrease in the protein expression of LC3-II/LC3-I in CTEPH rats was dependent on the concentration of TF, which ranged from 0 nM to 100 µM after 12 h. The protein expression of LC3-II/LC3-I at the same concentrations (0 nM, 1 µM, 10 µM, and 100 µM) was significantly lower in the CTEPH than in the ctrl groups after 12 h (*P* < *0.05*) (Fig. 5A). At the same time, we could see that there was a increasing trend in the protein expression of LC3-II/LC3-I in the ctrl group/CTEPH group depending on the TFPI concentration from 0 nM to 5 nM after 48 h. However, the protein expression of LC3-II/LC3-I was significantly lower in the CTEPH groups (TFPI concentration from 0 nM to 5 nM) than in the ctrl group at 48 h (*P* < *0.05*) (Fig. 5B). The expression of LC3-II/LC3-I may be negatively regulated by TF.

### Co-IP assays of FoxO1 and LC3 in PAECs

**Fig. 6:**
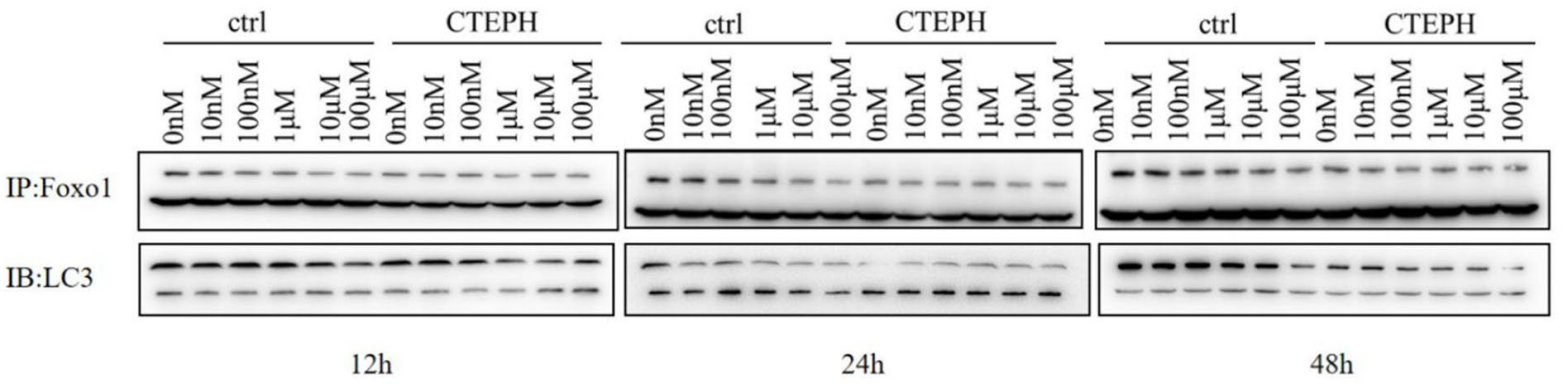
Co-IP assays of FoxO1 and LC3 in PAECs. Notes: In the ctrl group, PAECs from healthy SD rats were cocultured with TF; in the CTEPH group, PAECs from CTEPH rats were cocultured with TF

The results of co-IP assays for FoxO1 and LC3 are shown in Fig. 6. There were close interactions between FoxO1 and LC3 in the control and CTEPH groups at different doses and time points.

## Discussion

CTEPH is a type of pulmonary hypertension that is classified as Group 4 pulmonary hypertension [11]. CTEPH is a disease characterized by pathological changes in organized thromboembolic material and vascular remodeling with endothelial dysfunction [12]. Many factors contribute to the endothelial dysfunction observed in CTEPH patients, such as chronic thrombi, inflammation, infection and impaired angiogenesis [13]. After acute pulmonary thromboembolism, fresh thrombi without dissolution develop into chronic thrombi, which block the pulmonary artery and damage pulmonary arterial endothelial cells [11].

TF is positively correlated with venous thromboembolism, which is an initiator of the extrinsic coagulation pathway [14]. In a previous study, we found that TF was significantly increased in a CTEPH rat model and in patients with CTEPH [3, 4, 15]. In the present study, PAECs from CTEPH rats were cocultured with TF at different doses to simulate thrombus embolism. We next detected the effect of TF on FoxO1 and autophagy. In our previous study, the TF mRNA expression level was significantly greater in the CTEPH group than in the sham group, the FoxO1 mRNA and protein expression levels were lower in the experimental group than in the sham group, and the TF mRNA expression level was negatively correlated with the FoxO1 mRNA expression group [15]. In the present study, the protein expression of p-FoxO1 in PAECs gradually decreased in the control and CTEPH groups. Moreover, the protein expression of p-FoxO1/FoxO1 in the control and CTEPH groups gradually decreased depending on the TF concentration. Therefore, we can see that TF may play a key role in regulating the phosphorylation of FoxO1.

In our previous study, Beclin-1 and LC3B mRNA and protein expression levels were lower in the CTEPH rat group than in the ctrl group, and TF protein expression was negatively correlated with both Beclin-1 and LC3B protein expression [4]. We detected Beclin-1 and LC3-II/LC3-I expression in PAECs from rats and found that Beclin-1 and LC3-II/LC3-I expression also decreased with increasing TF concentration from 0 nM to 100 µM in this study. Therefore, the expression of autophagy-related proteins (Beclin1 and LC3-II/LC3-I) may be negatively regulated by TF concentrations, and TF may inhibit the autophagic activity of PAECs. Similarly, the protein expression of Beclin1 in the control and CTEPH groups decreased at 12 h, 24 h, and 48 h after treatment. The expression of LC3-II/LC3-I decreased after 12 h in the control and CTEPH groups. The expression of autophagy-related proteins (Beclin1 and LC3-II/LC3-I) may be negatively regulated by embolism, and autophagic activity may be impaired after embolism. However, the PAEC LC3-II/LC3-I ratio was not significantly different between 24 h and 48 h after treatment with different TF concentrations. This could be attributed to the duration of coculture, and we selected the most appropriate duration of coculture for the next study.

Wu et al. reported that FoxO1 mediates advanced glycation end products (AGEs)-induced vascular endothelial cell (EC) autophagic apoptosis through impairing autophagosome-lysosome fusion by inhibiting Atg14 expression [16]. Gong et al. also reported that autophagic flux was inhibited by ox-LDL and the Sirt1/FoxO1 pathway [17]. In this study, we detected the interaction between FoxO1 and LC3 in PAECs from the control and CTEPH groups at different doses and time points using co-IP assays and found that there was a close interaction between FoxO1 and LC3 in PAECs. Therefore, FoxO1 may mediate autophagy activity in PAECs.

A previous study revealed that p38α MAPK can stimulate FoxO1-S273 phosphorylation and that the p38α MAPK-pS273 signaling pathway is a potential therapeutic target for type 2 diabetes [18]. Zhao et al. reported that promoting FoxO1 and Nrf2 nuclear translocation may prevent lipotoxicity-induced endothelial injury and suppress inflammation by inhibiting the p38/NF-кB pathway [15]. In our study, we detected the expression of p38 MAPK in PAECs from the control and CTEPH groups and found that the protein expression of p38 in the control and CTEPH groups gradually increased as the TF concentration increased from 0 nM to 100 µM. Additionally, the protein expression of p38 was significantly greater after 24 h of treatment with the same concentration (from 0 nM to 100 µM) of the control or CTEPH treatment. Therefore, the expression of p38 may be positively regulated by TF and embolism.

In conclusion, the autophagic activity of PAECs from CTEPH rats was impaired. TF, FoxO1 and p38 MAPK play key roles in the autophagic activity of PAECs. TF may regulate autophagic activity through the p38 MAPK-FoxO1 pathway. However, we detected the expression of FoxO1 and p38 MAPK in PAECs treated with different concentrations of TF but not with respect to p38 MAPK, so we conducted further study.

## Data Availability

No datasets were generated or analysed during the current study.

## Abbreviations

FoxO1: Forkhead box transcription factor O-1
pFoxO1: Phosphorylated FoxO1
CTEPH: Chronic thromboembolic pulmonary hypertension
TF: Tissue factor
MAPK: Mitogen-activated protein kinase
PAECs: Pulmonary artery endothelial cells
EndMT: Endothelial mesenchymal transition
